# Cancer stem cells (CSCs), cervical CSCs and targeted therapies

**DOI:** 10.18632/oncotarget.10169

**Published:** 2016-06-19

**Authors:** Ruixia Huang, Einar K. Rofstad

**Affiliations:** ^1^ Department of Radiation Biology, Institute for Cancer Research, The Norwegian Radium Hospital, Oslo University Hospital, Oslo, Norway

**Keywords:** cervical cancer, cancer stem cells, cervical cancer stem cells, chemo/radio-resistance, cancer stem cell markers

## Abstract

Accumulating evidence has shown that cancer stem cells (CSCs) have a tumour-initiating capacity and play crucial roles in tumour metastasis, relapse and chemo/radio-resistance. As tumour propagation initiators, CSCs are considered to be promising targets for obtaining a better therapeutic outcome. Cervical carcinoma is the most common gynaecological malignancy and has a high cancer mortality rate among females. As a result, the investigation of cervical cancer stem cells (CCSCs) is of great value. However, the numbers of cancer cells and corresponding CSCs in malignancy are dynamically balanced, and CSCs may reside in the CSC niche, about which little is known to date. Therefore, due to their complicated molecular phenotypes and biological behaviours, it remains challenging to obtain “purified” CSCs and continuously culture CSCs for further *in vitro* studies without the cells losing their stem properties. At present, CSC-related markers and functional assays are used to purify, identify and therapeutically target CSCs both *in vitro* and *in vivo*. Nevertheless, CSC-related markers are not universal to all tumour types, although some markers may be valid in multiple tumour types. Additionally, functional identifications based on CSC-specific properties are usually limited in *in vivo* studies. Furthermore, an optimal method for identifying potential CCSCs in CCSC studies has not been previously published, and these techniques are currently of great importance. This article updates our knowledge on CSCs and CCSCs, reviews potential stem cell markers and functional assays for identifying CCSCs, and describes the potential of targeting CCSCs in the treatment of cervical carcinoma.

## CANCER STEM CELLS (CSCS)

Cancer stem cells (CSCs) are believed to be a small subpopulation of tumour cells that have properties of tumorigenesis, multilineage differentiation potential, self-renewal [1], slow cycling capacity [2] and tumorigenicity [3, 4]. In recent years, methods enabling tumorigenic cells and their progeny to be tracked and clearly observed *in vivo* have been developed, making the existence of CSCs increasingly more convincing [5–7].

CSCs are at a less-differentiated state than corresponding cancer cells. Similar to other stem cells, CSCs possess the capacity for asymmetrical division in addition to symmetrical division [8–10]. During asymmetrical division, CSCs divide into two different daughter cells, one of which copies the mother cell's entire genome, while the other has fewer features of stemness. Due to their ability to divide asymmetrically, CSCs possess the capacity for self-renewal and tumour initiation [10]. These properties of asymmetrical division and self-renewal enable CSCs to maintain dynamic control of their numbers, and tumours invariably consist of a mixture of CSCs and their diversely differentiated progeny, contributing to the significant phenotypic and functional heterogeneity of CSCs [11]. Due to their self-renewal and tumour-initiating properties, CSCs are believed to be the starting point for cancer and are thought to play key roles in cancer relapse and metastasis [12, 13]. As a result, CSCs have become a promising target for preventing cancer relapse and for vastly improving the survival of cancer patients [14–16].

CSCs are often dormant and remain in the CSC niche, which protect them from damage by any of the existing anti-tumour therapies [14, 17–19]. The CSC niche is a favourable environment for CSCs to achieve an optimal balance between self-renewal, activation and differentiation [20, 21]. In response to stress, CSCs are able to be “activated” and recruited into other tissues, where they differentiate and generate malignant cells [19]. Blagosklonny, M.V. noted that quiescent CSCs play a negligible role in advanced cancers that have a poor response to therapy and that only “activated” CSCs contribute to proliferation, progression and therapeutic failures. As such these cells should be targeted and eliminated [22, 23]. However, Gupta, G.B. and colleagues have discovered that cancer cells in various states were able to stochastically transit between states and generate a phenotypic equilibrium in breast cancer [24], indicating that immortal, quiescent CSCs, and even non-CSCs could be able to transit into proliferating CSCs when proliferating CSCs are eliminated [25–28].

Circulating tumour cells (CTCs), which are present in the blood, and disseminated tumour cells (DTCs), which are located in a secondary organ, are positively associated with tumour metastasis, relapse and poor survival [29–33]. Interestingly, CTCs and DTCs display the phenotypes of both CSCs and epithelial-mesenchymal transition (EMT) [34–37]. It is hypothesized that these CTCs and DTCs can evade immune targeting by undergoing EMT and losing their epithelial-related features. In this way, they achieve a more “de-differentiated” status and maintain more features of stemness while retaining their malignancy [33, 38]. In breast cancer, the proportion of CSCs in primary cancer is supposed to be less than 1% [39], whereas approximately over 50% of CTCs express EMT and CSC markers [40]. However, the relationship between CTCs, DTCs and CSCs is complicated and remains a topic of debate.

Cancer is known to be a heterogeneous disease [41–43]. First, there is inter-tumour heterogeneity, which involves different degrees of aggressiveness and clinical outcomes between patients who have the same tumour type. Second, there is intra-tumour heterogeneity, which involves biological and molecular differences between the tumour cells within the same tumour in a single patient [41, 44]. Cancer heterogeneity may be associated with the CSC content [45]. Histologically, tumours with a high percentage of CSCs may be poorly differentiated, undifferentiated or mixed tumours. As proposed by Weinberg RA et al., tumours are a heterogeneous mixture of CSCs that have mixed epithelial-mesenchymal phenotypes and non-stem cells that are epithelial [46]. By undergoing EMT, epithelial cells may acquire stem-like features [47–49]. However, only non-stem epithelial malignancies are attenuated or eliminated by existing anticancer treatments; by contrast, CSCs escape and survive [50]. As a result, to eliminate the cancer root, CSCs need to be specifically targeted and eliminated.

Multiple stem-cell specific markers and functional assays have been used to identify putative CSCs for both *in vitro* and *in vivo* studies. Some CSC-related markers are being developed as potential cancer therapy targets. However, it remains challenging to target CSCs because of their complex biology and instability [46, 51, 52]. A related problem is that the frequency and identity of tumorigenic cells varies from patient to patient, indicating that CSC markers identified in one tumour may not be sufficient for identifying CSCs in another tumour [53]. To date, no universal CSC marker for identifying CSCs in all tumours has been identified. Furthermore, CSCs reside in a CSC niche, which may preserve their phenotypic plasticity, protect them from the immune system and maintain their dynamic number and state [53–56], thereby making it more difficult to target them.

## CERVICAL CANCER STEM CELLS (CCSCS)

Cervical carcinoma is the most common type of gynaecological malignancy worldwide [57], and it is one of the leading causes of cancer death among females, especially in less developed countries [58]. In contrast to the traditional “clonal evolution” theory of carcinogenesis, which describes cervical carcinoma as a consequence of unlimited and uncontrolled cellular proliferation, there is heterogeneity in cervical carcinoma. Intra-tumour genetic heterogeneity in cervical carcinoma is associated with a poor chemo/radio-therapy response [59], lymph node metastasis and pelvic recurrence [60]. One explanation for the heterogeneity in cervical carcinoma is the existence of CCSCs. Because of the asymmetrical division of CCSCs, cervical carcinoma tissue consists of diversely differentiated carcinoma cells. Furthermore, CSCs have recently been found to be capable of transdifferentiation into vascular endothelial cells and other tumour-associated stromal cells [61], which may also contribute to tumour heterogeneity.

**Figure 1:**
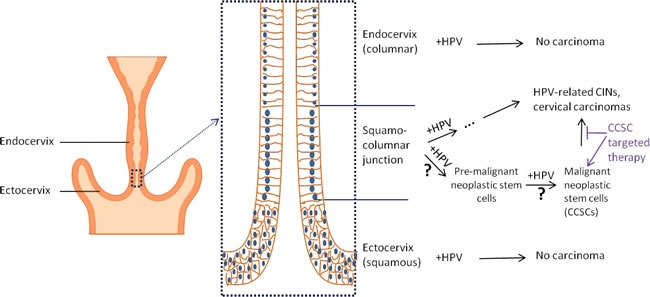
Illustration of cervical carcinogenesis and CCSCs Carcinogenic HPV infection has a causal relation with cervical carcinogenesis. However, when the cervix is infected with carcinogenic HPV, HPV-related CINs and cervical carcinomas are usually generated within a specific cell population that is located in the ectoendocervical squamocolumnar (SC) junction of the cervix. They are typically not generated in the columnar cells located within the endocervix and squamous cells within the ectocervix. The HPV-related CINs and cervical cancers maintain the genetic profile of the junction cells, indicating their cellular hierarchy. Progenitor cells located in the junction area infected with carcinogenic HPV are likely to become pre-malignant neoplastic stem cells that can propagate malignant neoplastic stem cells (CCSCs), which propagate cervical carcinoma clones. Therapeutically targeting these cells may prevent the propagation of HPV-related CINs and cervical carcinomas.

Cervical carcinoma is known to have a causal relationship with specific human papillomavirus (HPV) strains [62]. However, not all cervical epithelial cells infected with carcinogenic HPV will generate cervical carcinoma. After infection with carcinogenic HPV, cells located in the transition area between the endocervix and exocervix, which is known as the squamo-columnar (SC) junction, more easily lead to cervical intraepithelial neoplasia (CIN) and carcinogenesis [63, 64]. Studies have shown that approximately 90% of CIN3 and cervical cancer arise within or very near the SC junction [64]. These specific SC junction cells exhibit unique morphology and gene profiles, which distinguish them from the adjacent endocervical and ectocervical epithelium. For instance, they express SC junction-specific markers, keratin 7 (Krt7), anterior gradient 2 (AGR2), cluster differentiation 63 (CD63), matrix metalloproteinase 7 (MMP7) and guanine deaminase (GDA). Intriguingly, these junction-specific markers are also expressed in carcinogenic HPV-associated CINs and carcinomas, including both squamous cell carcinomas and adenocarcinomas, indicating that multiple cervix malignancy subtypes are derived from the SC junction cells [63, 65]. Similarly, ovarian carcinoma originates in the transition area between the ovarian surface epithelium, mesothelium and tubal epithelium, and these junction cells have long-term stem cell properties *ex vivo* and *in vivo*, as determined by serial sphere generation and long-term lineage-tracing assays. As a result, the junction area has been considered to be an important cancer stem cell niche in which ovarian cancer originates [18]. We hypothesize that cervical carcinoma develops from stem-like cells in the transition area of the cervical opening that are infected with carcinogenic HPV and that the junction/transition area may be a possible niche for CCSCs (Figure 1).

Cervical cancer treatment includes surgery, chemotherapy and radiation therapy, and these approaches have improved carcinoma survival. Cisplatin-based chemotherapy is a commonly used cervical cancer therapy [66]. However, some CCSCs that were identified by multi-marker and functional examinations are resistant to cisplatin-based chemotherapy [67] and radiation therapy [68], which is in agreement with the chemo/radio-resistance that is generally observed with CSCs.

Considering the tumorigenic potency of common CSCs, CCSCs may be the route of cervical carcinogenesis and lead to distant metastasis. According to this theory, therapeutic treatment that specifically targets CCSCs may prevent the generation of new tumours. Such a treatment approach would offer a critical tool for preventing distant metastasis, tumour relapse and chemo/radio-resistance. With the combination of CCSC-targeted therapy and traditional anti-cancer therapy, cervical cancer might someday be curable. To achieve this goal, the first requisite and most challenging problem is how to specifically identify and target CCSCs as part of cancer treatment.

However, just as CSCs have a CSC niche, CCSCs may also reside in their own niche, which may differ from the tumour microenvironment. The CSC niche is not well understood, and the characteristics, cellular communication and molecular profile of the CCSC niche are even more obscure, making the identification of CCSCs even more challenging. The existing approaches for identifying CCSCs primarily depend on the expression of specific stem cell markers in malignant cells and their stem-like functional properties.

## STEM CELL MARKERS FOR CCSCS

Stem cell markers are widely used to identify CSCs for both *in vitro* and (especially) *in vivo* studies because of the practical limitations of functional assays. Therefore, targeting CSC-specific markers is one of the most promising and easily achievable approaches for identifying CSCs, even though molecular assays are not sufficient to fully define CSCs [69]. Stem cell markers for CCSCs have been accumulating, and novel markers are being identified. We reviewed and summarized the markers for CCSCs that are currently used or are potential candidates in CCSC studies (Table 1). However, as with the heterogeneity of CSCs, CCSC markers vary from tumour to tumour. Consequently, cervical cancer cells that express a single stem cell marker do not always qualify as CCSCs.

**Table 1:** CCSC markers that are currently in use

marker	Cell lines	Establishment of stem-like cells	Stem-like properties	Clinical associations
ABCG2	SiHa, CaLo, C-33A	SP	High colony forming efficiency, multilineage differentiation, asymmetrical division [78]	
ALDH1	SiHa [67, 84], C33A, CaSki, HT-3 [67]	ALDH1+ cells by FACS using Aldefluor staining	High cell proliferation, migration, sphere forming efficiency [84], limiting dilution tumorigenicity, multilineage differentiation, asymmetrical division, self-renewal, highly expressing OCT4, NANOG, KLF4 and BMI1 [67]	Poor survival [83]
CD133	Hela	SP	High cell proliferation, self-renewal, chemo/radio-resistance, limiting dilution tumorigenicity, multilineage differentiation, anti-apoptosis, highly expressing OCT4, ABCG2, SOX2 [98–100]	
CD49f	HeLa, SiHa, Ca Ski, C-4 I	Tumorigenic spheroids	Limiting dilution tumorigenicity, self renewal, highly expressing stem cell markers and EMT markers, radioresistance [68]	
OCT4	HeLa, SiHa	OCT4 over-expressing cells by plasmid transfection	Enhanced tumorigenicity, anti-apoptosis [113]	Poor differentiation [111], lymph node metastasis [111, 113], radioresistance, poor survival [112]
OPN	HeLa, SiHa	OPN over-expressing cells by plasmid transfection	Enhanced tumor growth, stimulating CD44 phosphorylation and CD44-dependent MAPK and NF-κB activation [123]	Hypoxic radiation resistance and poor survival [121]
SOX2	SiHa, C33A	SOX2 over-expressing cells by plasmid transfection and cell sorting by SOX2 antibody by FACS	Differentiation, self-renewal, enhanced tumorigenicity, highly expressing stem cell markers OCT4, ALDH1, BMI1 and EMT-related markers vimentin, snail, β-catenin [134]	Higher SOX2 expression in cervical carcinoma than normal cervix [129–131], in high grade of dysplasia than low grade of dysplasia [133], enhanced radioresistance, poor survival [112]

## ABCG2

ATP-binding cassette sub-family G member 2 (ABCG2), also known as breast cancer resistance protein (BCRP), is a drug efflux membrane transporter of the ATP-binding cassette (ABC) family. ABCG2 pumps out a wide variety of chemical compounds from cells and plays a major role in multi-drug resistance in a number of cancer types [70]. It is also a determining molecular marker in a side population (SP) phenotype, which is considered to be a characteristic feature of CSCs [71, 72]. Therapies that target and block the function of ABCG2, such as Axitinib [73] and Icotinib [74], could enhance cellular sensitivity to chemotherapy. ABCG2 has thus become a CSC marker and a potential target for cancer treatment [75]. The expression and activity of ABCG2 may interact with various lipid compounds, especially those residing in close proximity within the plasma membrane [76]. In cervical cancer, the redox sensing factor Nrf2 may play an important role in the transcriptional regulation of ABCG2, and cells with upregulated Nrf2 and ABCG2 exhibit stem-like characters, including infinite cell proliferation, longevity and prevention of apoptosis [77]. Three commercial cell lines, SiHa, CaLo, and C-33A, were sorted using fluorescence-activated cell sorting (FACS) to obtain SP and non-SP (NSP) cells. The SP cells had high ABCG2 expression and colony forming efficiency, as well as the capacity to generate both SP and NSP cells, suggesting that ABCG2 plays a pivotal role in maintaining cell stemness [78].

## ALDH1

Aldehyde dehydrogenase 1 (ALDH1) is a metabolic enzyme that is localized in the cytoplasm and catalyses the dehydrogenation of aldehydes. ALDH1 is also associated with the tumorigenic cell fraction, capacity for self-renewal and tumorigenesis [79], especially in breast cancer [80, 81]. Additionally, the presence of ALDH1-positive CSC-like cells in primary breast cancer is associated with the successful establishment of patient-derived xenografts [82].

ALDH1 expression in cervical carcinoma patient tissues is an independent risk factor that indicates a poorer survival probability [83]. High ALDH1 expression in cervical cancer cells is associated with a high rate of cell proliferation, sphere formation, migration and tumorigenesis [84], indicating that it acts as a stemness factor in cervical cancer. To determine whether ALDH could serve as a specific marker of CCSCs, ALDH-high and ALDH-low cells were sorted from 4 cervical cancer cell lines and 5 primary tumour xenografts using FACS and were then examined for the presence of CSC characteristics. ALDH-high cells had a higher tumorigenicity potential *in vivo* than ALDH-low cells, and they could divide into both ALDH high and low cells *in vitro* and *in vivo*, thereby establishing a cellular hierarchy and enhancing self-renewal and differentiation potentials [67].

## CD133

CD133 is a pentaspan transmembrane glycoprotein (120 kDa) encoded by the prominin 1 (PROM1) gene in humans [85]. CD133 has been extensively used as a CSC marker in many tumours [86], including brain [87], breast [88], colon [89], liver [90, 91], lung [92], melanoma [93, 94] and ovarian [95–97] cancers, although there are limited reports on the relationship between CD133-positivity and the stem-like characteristics of tumour cells. In cervical cancer cells, it was found that the SP cells that were sorted using FACS from the HeLa line, which displays the stem-like probabilities of proliferation, differentiation, self-renewal, chemo/radio-resistance and tumorigenicity, had high CD133 expression compared with the NSP cells [98, 99]. As a result, CD133 may serve as a specific CCSC marker in CSC-targeted therapy. Other studies have also shown that established cervical stem-like cells express CD133 and other CSC-related markers and exhibit radiation resistance [68, 100].

## CD49F

CD49f is a cell surface protein that is encoded by the integrin alpha 6 (ITGA6) gene. It is highly expressed in human embryonic stem cells (ESCs) [101], mesenchymal stem cells (MSCs) [101, 102] and haematopoietic stem cells (HSCs). In MSCs, CD49f enhances multipotency and maintains stemness by directly regulating thetranscription factors POU class 5 homeobox 1 (POU5F1, OCT4) and SRY-box2 (SOX2) [101]. HSCs can be purified from mobilized peripheral blood cells based on CD49f expression [103]. CD49f and CD44, a stem cell marker, are transcriptionally upregulated by Y-box binding protein-1 (YB-1) in breast cancer cells, enhancing the stem-like properties of these cells, which include self-renewal, colony forming efficiency and drug resistance [104]. CD49f is also involved in maintaining stem-like features in multiple cancer types, including gastric, colon, and prostate cancer [105–108]. Furthermore, CCSC models have been established in cell line-developed tumorigenic spheroids that are resistant to radiotherapy, and these CCSC models have high CD49f expression [68].

## OCT4

OCT4, also known as OCT3 and OCT3/4, is a transcription factor that is expressed by the POU class 5 homeobox 1 (POU5F1) gene in humans. It plays a key role in embryonic development and the maintenance of stem cell pluripotency [109, 110]. OCT4 is over-expressed in cervical cancer tissues compared to adjacent normal tissues [111], and the over-expression of OCT4 in cervical cancer cells is associated with a low-differentiation grade of cervical cancer cells and positive lymph node metastasis [111]. Clinical data have shown that high OCT expression is positively associated with radiotherapy resistance, and OCT expression has been found to be an independent risk factor for cervical cancer patient survival [111, 112]. Furthermore, an *in vitro* study demonstrated that OCT4 promotes tumorigenesis and inhibits cancer cell apoptosis [113].

At least three isoforms (OCT4A, OCT4B and OCT4B1), which are produced by alternative splicing, were discovered, and they may play different roles in stem cell biology [114, 115]. Among the three identified isoforms, nuclear OCT4A is the most widely studied and has been recognized as a key factor in regulating pluripotency [115, 116]. In cervical carcinoma, both nuclear OCT4A and cytoplasmic OCT4B have been reported to be over-expressed [117]. OCT4A is responsible for the maintenance and self-renewal of CCSCs, and it may serve as a CCSC marker, while cytoplasmic OCT4B may work with OCT4A to regulate cervical carcinoma progression by inducing angiogenesis and EMT [117].

## OSTEOPONTIN (OPN)

Osteopontin (OPN), a chemokine-like extracellular matrix protein, is secreted by malignant cells and tumour stromal cells. OPN is a key mediator of tumour cell migration and metastasis [118, 119]. OPN was primarily found to be an endogenous hypoxic marker because it is upregulated by hypoxia [120] and tends to bind to the hypoxic regions of tumour tissues. The over-expression of OPN predicts hypoxic radiation resistance and poor survival in human cervical cancer [121]. OPN may also induce tumour angiogenesis by modulating HIF1α-dependent VEGF expression in response to hypoxia [122]. OPN over-expression in a murine xenograft model of human cervical cancer enhanced tumour growth; conversely, OPN silencing, mediated by short hairpin RNA, blocked this effect [123]. OPN could also be detected in the blood, and an elevated serum OPN level predicts poor survival in cervical cancer patients [124].

OPN expression in hepatocellular carcinoma cell lines is associated with high SP fractions, spheroid formation and tumorigenicity rates in immunodeficient mice [125]. OPN can bind to CD44 receptor family members and regulate tumour cell fate through OPN-CD44 signalling [125]. OPN regulates CD44-mediated p38 phosphorylation, affecting downstream genes, nuclear factor kappa-light-chain-enhancer of activated B cells (NF-κB) and NF-κB-dependent expression of furin, which are involved in the response of human papilloma virus (HPV) [123]. OPN-CD44 signalling may enhance cancer stem-like features [126], and the OPN-mediated self-renewal capabilities may be suppressed by the reduction in NF-κB expression [125].

## SOX2

SOX2 is a key transcription factor that is involved in embryonic development and plays a critical role in determining stem cell fate [127, 128]. There is significantly higher nuclear SOX2 expression in cervical carcinoma than in normal cervix tissues [129–131]. Furthermore, cervical cancer with high SOX2 expression is more poorly differentiated [130], indicating that SOX2 might be a marker for undifferentiated cervical cancer. Both *in vitro* and *in vivo* studies have shown that cervical cancer cells with SOX2 over-expression have increased cell proliferation [132], clonogenicity, and tumourigenicity [129]. Oncogenic virus HPV-positive cervix cells that generate a higher grade of dysplasia often have higher SOX2 expression than those that generate a lower grade of dysplasia [133]. This indicates that SOX2 plays an important role in early tumour initiation. Moreover, cervical squamous cancer patients with high SOX2 expression in tumour cells have enhanced radiation resistance [112]. The cervical cancer cell lines, SiHa and C33A, were transfected with a plasmid containing the human SOX2 gene, and were sorted using a SOX2 antibody and FACS. The SOX2-positive population expressed higher levels of stem cell-related genes, OCT4 and ALDH1, and EMT-related genes, indicating that CCSCs were more likely to be SOX2 positive cells [134]. Moreover, the SOX2 positive population showed higher probabilities for differentiation, self-renewal and tumourigenicity [134], which further confirms the role of SOX2 as a practical CCSC marker in stem-cell functional examinations.

## OTHER POTENTIAL MARKERS FOR CCSCS

There are a number of specific markers for CSCs that could potentially serve as CCSC markers, although little evidence supports their use for this purpose. These include the BMI1 proto-oncogene, polycomb ring finger (BMI1) and Kruppel-like factor 4 (KLF4). BMI1 is a core transcription factor involved in the regulation of EMT and CSC self-renewal, and it integrates multiple signalling pathways, such as TWIST1 [135, 136]. KLF4, as a well-known member of the KLF family, is one of the four factors that can reprogram adult fibroblasts into induced pluripotent stem cells. The significance of KLF4 in CSC regulation has recently increased [137–139]. BMI1 and KLF4 expression levels were found to be elevated in ALDH-high cervical cancer cells that display high stem-like features compared to the ALDH-low cell population [67], indicating that BMI1 and KLF4 may be CCSC markers.

## CD44

CD44 has been accepted as a CSC marker in a number of tumours, including gastric cancer [140], colorectal cancer [141–143], glioma [126], head and neck cancer [144, 145] and breast cancer [146]. Many functionally distinct isoforms could be encoded by the CD44 gene because of the complex alternative splicing of transcripts, and these isoforms may participate in different oncogenic signalling pathways and play different roles in tumour progression [147, 148]. Among these splicing variants, the smallest isoform, CD44s, which lacks all variant exons, is the standard CD44 [149] and the one associated with CSCs [148, 150, 151]. However, an immunohistochemical (IHC) study demonstrated that CD44s could be largely expressed in both normal cervix and cervical cancer, and soluble CD44s in serum was also found in both normal and invasive cervix [152]. Sorted SP and NSP cells from the HeLa line, which showed significant differences in many stem-like characteristics, did not have significant differences in their CD44 expression [99]. To date, the evidence for CD44 as a specific CCSC marker remains insufficient, although it is widely used as a general CSC marker in many tumours.

## C-KIT

A proto-oncogene, c-Kit, which is also known as tyrosine-protein kinase KIT or CD117, is a transmembrane cytokine receptor expressed on the surface of haematopoietic stem cells and other cell types. It is normally phosphorylated and activated by binding to the KIT ligand, which is also called stem cell factor [153]. It is a widely used stemness marker for recognizing cancer stem cells in various tumour types, including ovarian cancer [154–156], endometrial cancer and osteosarcoma [155, 157, 158]. In HPV-associated cervical cancer, SCF-activated c-Kit may activate interleukin-2 receptor betagamma signalling in the absence of IL-2, promoting T cell proliferation [159]. Unusual expression of c-Kit was found in cervical squamous cell carcinoma without c-Kit gene amplification [160], and the tumours with this unusual c-Kit expression had a high DNA methylation ratio and hypermethylation of the c-KIT promoter [161], suggesting that the c-Kit expression may be regulated by DNA methylation. However, the evidence that c-Kit serves as a stem cell marker for cervical cancer is not yet comprehensive.

## NANOG

NANOG homeobox (NANOG) is another transcription factor, in addition to SOX2 and OCT4, which is highly expressed in human ESCs. NANOG plays an essential role in ESC maintenance of pluripotency and the regulation of proliferation and asymmetric division [162, 163]. NANOG has been broadly reported as a CSC marker that regulates self-renewal and tumorigenesis in many tumour types [102, 164–172]. The human form of NANOG has 11 pseudogenes, of which NANOGP8 encodes a functional protein product with only 3 amino acids that differ from the product of NANOG [173]. An IHC study clarified the expression of NANOG in patients with cervical cancer and cervical dysplasia, and its expression was found to be significantly higher in cervical cancer than in cervical dysplasia and higher in cervical dysplasia than in normal cervical epithelia [174], supporting the role of NANOG in carcinogenesis and cervical carcinoma progression. Interestingly, according to the IHC study, in cervical cancer cells and surrounding stromal cells, NANOG was frequently observed in the cytoplasm, instead of the nucleus, where it is found in many other cell types [175]. The cytoplasmic NANOG that is expressed in stromal cells may promote cervical cancer progression [175].

## FUNCTIONAL ASSAYS FOR IDENTIFY-ING CCSCS

As in normal stem cells, functional assays based on stemness-specific properties are the gold standard criteria for identifying CSCs. Functional verification of CCSCs is based on the same rules that are used for CSCs. They include clonogenic activity in soft agar, sphere-forming efficiency in non-adherent cultures, examination of self-renewal and the differentiation potential, and tumorigenic capacity according to the limiting dilution tumorigenicity assay [69]. Asymmetrical cell division is a characteristic and marked manifestation of self-renewal and is assayed by checking whether the “purified” test cells can divide into two populations, one that mimics the parent cell and another with different or more differentiated features. In some cases, the presence of an activated self-renewal pathway is thought to be typical, e.g., PTEN/Akt/ β-catenin signalling and PTEN/Akt/PI3K signalling [176, 177]. Anti-apoptosis may also be a discriminating characteristic of CSCs [178]. The presence of chemo-resistance and radiation resistance also helps characterize CSCs [179, 180]. Nevertheless, all CSC-specific functional assays have been performed *in vitro*, and it remains challenging to examine the CSC-related functions of potential CSCs *in vivo*. Therefore, the role of CSC markers in identifying CSCs *in vivo* is currently emphasized, although they are not the standard criteria and are insufficient for specifically identifying CSCs.

SP is widely recommended as another test for identifying CSC populations [181–185]. The SP discrimination assay is a method that uses flow cytometry to detect stem cells and CSCs based on the dye efflux properties of the ABC family of transporter proteins expressed within the cell membrane [186]. The sorting method based on SP has been considered to be simple and effective in cancer stem cell research [187]. A human cervical cell line, HeLa, was sorted using FACS into SP and NSP cells, and the SP population had higher expression of a CCSC marker, CD133, and displayed most of the classic CSC characteristics, such as increased proliferation, self-renewal, differentiation potential, tumorigenicity and chemo/radio-resistance [98, 99]. However, the SP phenotype is not exclusive to stem cells and is not universal in all cancer types [188]. The procedure for SP population detection is continually being optimized to achieve more specific and sensitive results [186].

## NOVEL STRATEGIES AND CHALLEN-GES FOR CSC TARGETED THERAPY

To improve the current treatment of cancer and prevent cancer relapse, CSC-targeted therapy has been intensively studied in recent years. However, to date, studies on specific CCSC targeted therapies are very limited. We will generally discuss CSC targeted therapies, which may hint at potential strategies for CCSC targeted therapies. CSC-specific markers and signalling pathways have been largely used as therapeutic targets, and a variety of CSC targeting strategies are being studied.

The dual-targeting strategy has been proposed to target CSCs in recent years [189]. VS-5584, as a potent and selective dual inhibitor of mTORC1/2 and class I PI 3-kinases (PI3K), specifically targets human CSCs and inhibits their tumour initiating capacity, as demonstrated in murine xenograft models with human breast and ovarian cancers [190]. More studies on dual-targeting therapy that could be used to target CSCs are under investigation. For instance, at the American Association of Cancer Research (AACR) 106^th^ Annual Meeting 2015, dual targeting of delta-like ligand 4 (DLL4) and programmed death 1 (PD1) was demonstrated to be a promising cancer therapy [191]. However, there is insufficient information to make a conclusive statement about the therapeutic potential of the dual-targeting strategy, and no studies have yet reported on the use of dual-targeting to treat CCSCs. More studies on dual-targeting are warranted.

CSC targeting with nanoparticles (NPs) is another novel, possibly effective therapeutic approach that has been a topic of recent, exciting investigations [192–195]. NP-enabled therapies have been designed to inhibit stem cell-related functions by targeting stem cell-specific signalling pathways (e.g., Wnt/β-catenin [196], Notch [197] and reactive oxygen species (ROS) signalling [198]) and/or CSC-specific markers (e.g., CD44 [199, 200]) that are critically involved in maintenance of cell stemness [201]. Kumar P et al. proposed an approach for targeting endothelial cells in the cancer stem cell niche using a twin NP of iron coated with gold [202]. Gold NPs that were further conjugated with sophorolipids were found to be effective in the treatment of human glioma stem cells [203]. NP-mediated photothermal therapy is effective for both breast cancer stem cells and non-stem cancer cells [204]. A hybrid NP of bioactive quinacrine and silver was also shown to enhance cytotoxicity and inhibit oral stem cells *in vitro* [205]. However, NP-enabled therapies remain far from an ideal CSC-specific targeting therapy, especially because sensitive, specific markers or an equal combination of different markers and distinctive CSC signalling pathways have not yet been characterized for each tumour type.

Although studies on CSC-targeting therapies have increased in recent years, there remain some limitations that are not easy to overcome. CSCs are typically present at very low levels in tumours, accounting for only approximately 0.1-10% of tumour cells [206]. Furthermore, most of the currently available information on CSC targeting therapy is largely inspired and influenced by the biological characteristics of normal stem/progenitor cells, such as their discriminating surface markers and specific signalling pathways. As a result, CSC-targeted therapy may damage normal stem/progenitor cells and block the regeneration of normal tissues, causing tissue or organ dysfunction.

## CONCLUSIONS

Cancer is a heterogeneous disease that consists of a small subpopulation of CSCs with mixed epithelial-mesenchymal phenotypes and non-stem cells with an epithelial phenotype. CSCs have become promising targets for cancer treatment due to their capacity for self-renewal and tumorigenicity. CSC-specific markers and functional assays are widely used to identify CSCs. However, because of their practical limitations, functional examinations for CSCs are not as commonly used as CSC-specific markers in *in vivo* studies.

CCSCs, which can generate multiple tumour cells, seed distant metastases and promote tumour recurrence, are becoming promising targets for treating cervical carcinogenesis. HPV-associated cervical carcinoma may arise from the HPV-infected cells in the SC junction area, which may act as the CCSC niche. Several markers, such as ABCG2, ALDH1, CD133, CD49f and SOX2, were identified as CCSC-specific markers in well-known cervical cancer cell lines based on their stem-like functional probabilities *in vitro*. The targeting of various stem cell related-markers and signalling pathways may offer a novel strategy for CSC-targeted therapy, such as through dual-targeting and NP-enabled therapies. Challenges for CSC targeted therapy remain, including potential damage to normal stem/progenitor cells.
